# Short- and mid-term clinical outcomes of harmonic scalpel-assisted no-touch technique of the saphenous vein grafts harvesting in coronary bypass grafting

**DOI:** 10.1186/s13019-025-03823-x

**Published:** 2025-12-30

**Authors:** Ge Zhu, Su Wang, Chenjun Han, Qiang Liu, Jian Zhou, Wangfu Zang

**Affiliations:** Department of Cardio-Thoracic Surgery, Shanghai Tenth People’s Hospital, School of Medicine, Tongji University, Shanghai, China

**Keywords:** Coronary heart disease, Coronary artery bypass grafting, Harmonic scalpel, Great saphenous vein

## Abstract

**Background:**

The “No-touch” technique has demonstrated efficacy in enhancing both short-term and long-term patency of great saphenous vein grafts (SVG) for coronary heart disease (CHD) treatment. Nevertheless, its widespread use is constrained by the method’s limitations. Therefore, we modified it by using a harmonic scalpel to harvest SVG and verified its patency rate.

**Methods:**

156 patients who underwent coronary artery bypass grafting (CABG) were consecutively recruited between November 2018 and July 2019. Patients were allocated to 2 groups of 78 each, according to two SV harvesting techniques (modified no-touch [M group] and conventional technique [C group]). SVG samples were taken for pathological examination. This study was conducted according to the guidelines of the Declaration of Helsinki and was approved by the Ethics Committee of Shanghai Tenth People’s Hospital (ChiCTR1800018433). All patients underwent follow-ups for at least 2 years.

**Results:**

The modified no-touch technique reduced graft acquisition time (*P* < 0.001) and the pulsatility index (PI) (*P* < 0.001). No difference was detected in the average flow of grafts and procedural complication rate. At 24 months, the left ventricular ejection fraction (LVEF) was higher in the M-group (*P* < 0.001).

**Conclusions:**

The use of the harmonic scalpel to harvest SVG is safe and effective, and may contribute to better postoperative cardiac function recovery, as reflected by satisfactory short- and mid-term outcomes.

**Supplementary Information:**

The online version contains supplementary material available at 10.1186/s13019-025-03823-x.

## Background

Cardiovascular diseases remain the riskiest threat to human health, among which coronary heart disease (CHD) is dominant in epidemiology [1]. Coronary artery bypass grafting (CABG) remains the standard treatment for complex and severe anatomical stenosis of multiple vessel diseases because the long-term patency rate of bypass conduits is higher than that of stents [2, 3]. Mainland China and Hong Kong completed 559,862 cases of CABG in 2021, accounting for 21.5% of total cardiac surgeries according to statistics [4].

Currently, the materials used in bypass grafting include internal mammary artery (IMA), radial artery (RA), gastroepiploic artery (GA), and great saphenous vein (SV) [5]. While arterial grafts exhibit higher 10-year patency rates (85–91%), the SV remains widely used for its accessibility, despite a 50% long-term patency rate [6, 7]. Although mortality has been shown to be lower with the radial artery as the second graft for CABG [8], a recent study with a nearly 18-year follow-up showed no difference in survival between patients who used SV and RA [9].

Venous graft failure often stems from intraoperative vascular injury caused by traction, high-pressure perfusion, or arterial pressure exposure post-transplantation [10, 11]. These factors induce endothelial hyperplasia, smooth muscle cell migration, extracellular matrix deposition, and subsequent intimal thickening or atherosclerosis [10, 11]. Finally, these changes provoke graft stenosis [10–12]. Although the “No-Touch” technique reduces mechanical trauma by preserving perivascular tissue, its complexity limits clinical adoption [13]. In order to retain the advantages of the No-Touch technique and optimize the process of SV graft acquisition, combined with existing techniques, we developed a method of vein harvesting called the modified “no-touch” harvesting technique (MNT), using a harmonic scalpel to harvest veins with a pedicle of surrounding tissue. We conducted the following trials with the purpose of comparing important perioperative clinical indicators and follow-up results between this modified NT technique and conventional methods of graft harvesting.

## Methods

### Study design

This randomized and longitudinal trial was initiated in 2018 at the Department of Cardio-thoracic Surgery, Tenth People’s Hospital. One hundred and fifty-six patients who met the criteria for undergoing CABG between November 2018 and July 2019 were allocated to 2 groups of 78 each according to two SV harvesting techniques (M-group and C-group). Patients were randomly assigned to either M-group or C-group in a 1:1 ratio using a computer-generated random number sequence. In all groups, the veins were harvested and used in the cases by the same surgeon. This study was conducted according to the guidelines of the Declaration of Helsinki and was approved by the Ethics Committee of Shanghai Tenth People’s Hospital (ChiCTR1800018433). All patients signed their informed consent.

Patients enrolled in our study had to meet all the following criteria: (1) less than 80 years old; (2) left ventricular ejection fraction (LVEF) > 35%; (3) no evidence of varicosity of the great saphenous vein; (4) non-emergency surgery. Exclusion criteria were unstable angina, serum creatinine > 177 µmol/L, contraindications to oral anticoagulants and antiplatelet agents, coagulopathy, combined procedure, redo CABG, and severe peripheral vascular disease.

## Harvesting techniques

### (1) Preoperative preparation

Preoperative ultrasonography of the lower limbs was performed using a Philips EPIQ 7 C ultrasound system (Philips Healthcare, USA) equipped with a 7–12 MHz linear array probe. The procedure was used to assess the course, diameter, and branches of the saphenous vein, and to mark its trajectory on the skin prior to incision.

### (2) Intraoperative protocol

Systemic heparinization was administered before the vein was fully exposed for harvesting to avoid thrombogenesis during the harvesting process. A longitudinal lower limb incision was made for SV exposure using an open technique to minimize traction.

### C-Group

The grafts were skeletonized using scissors, and side branches were secured with titanium hemostatic clips. The vein was removed from the leg immediately after dissection and checked for leakage by manual distension with saline. The distension pressure was monitored using a syringe connected to a pressure gauge, and the measured pressure typically reached at least 300 mmHg during expansion.

### M-Group

**Fig. 1:**
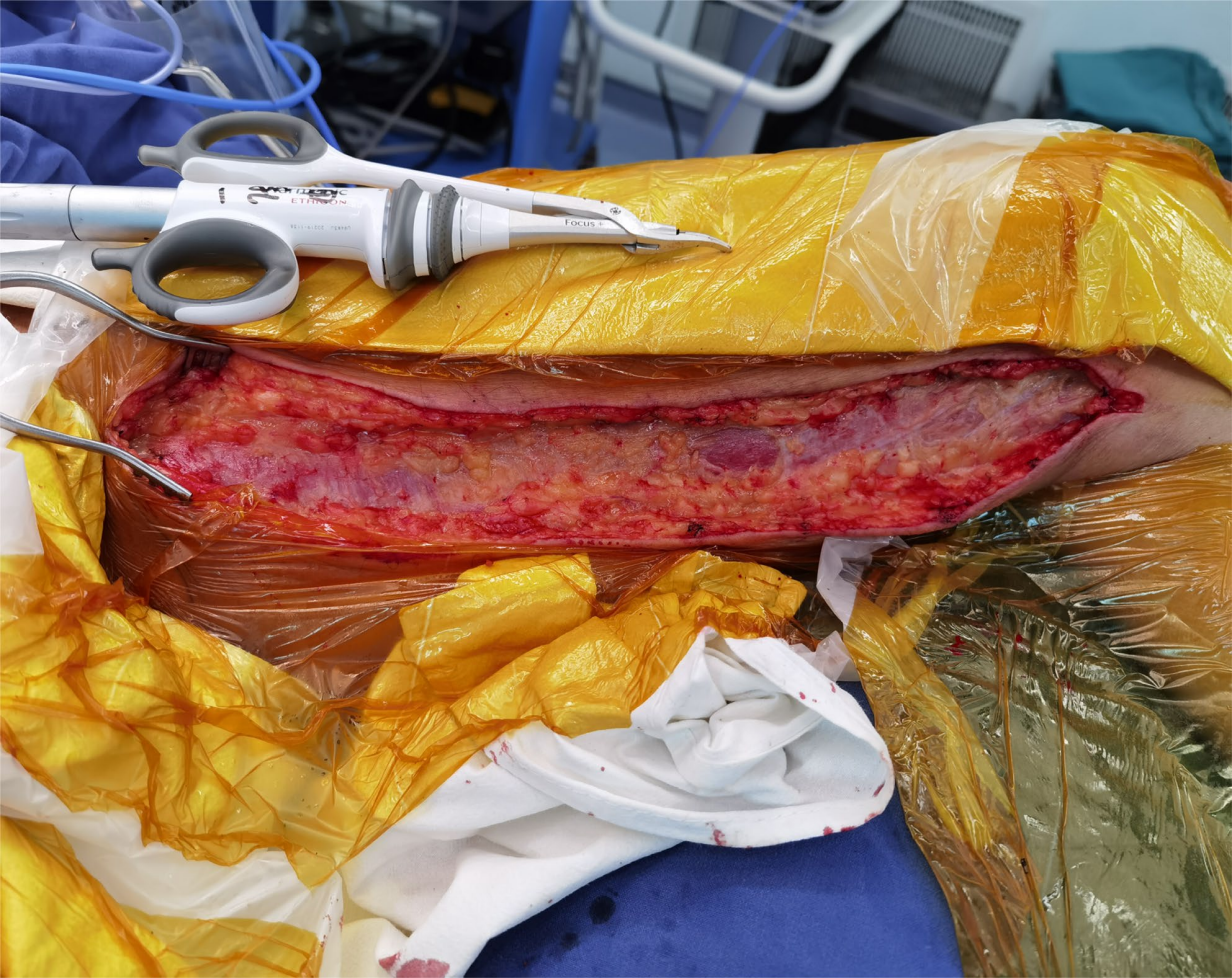
Harmonic scalpel for MNT technique. This photograph shows the harmonic scalpel used for harvesting SV. The harmonic scalpel can quickly close small vessels in the tissues around SV through high temperature

A harmonic scalpel (Fig. 1) was used to seal branches without manual dilation. Vascular patency was verified by saline injection. Then the larger-diameter branches or those deemed to require reinforced closure of the stump were ligated with titanium hemostatic clips. The vein was then harvested together with a pedicle of surrounding fat tissues and left in situ until the proximal anastomosis to the aorta was ready to be performed. After removal, the vein was transplanted to the aorta immediately and observed for blood leakage under arterial pressure. In cases of minor leakage, the site was repaired under direct vision using 7 − 0 Prolene sutures. If a distinct branch was present, it was ligated or closed with titanium hemostatic clips.

## Anastomosis strategies

We used sequential anastomosis with a single aortic anastomosis stoma.

## Intraoperative graft’s flow measurement

Using a surgical flowmeter (Norway Medi-stimQuickFit) to measure the flow and pulsatility index (PI) at mean arterial pressure (MAP) of 90 mmHg.

## Temperature measurement

Temperature around the grafts was measured with an infrared thermometer (FLIRONE^®^PRO, P/N 435-0007−01, USA) during the harvesting process.

### Histomorphology

The harvested SVG were fixed with 4% paraformaldehyde, paraffin-embedded, and sectioned into 5 μm-thick slices. Hematoxylin and eosin (H&E) were used for characterizing the morphological changes according to standard staining protocols.

## Immunohistochemistry

Immunohistochemical analysis was performed on routinely prepared 5 μm-thick vein sections. The integrity of the endothelium and wall structures of the SV was estimated using antibodies targeting CD34 and Collagen I.

## Follow-up

All patients underwent follow-ups for at least 2 years. One year after surgery, patients underwent coronary artery CT angiography (CTA) to observe graft patency results and echocardiography to evaluate cardiac function. The end-points included death (cardiac and non-cardiac), lower extremity edema, lower extremity numbness, angina pectoris, LVEF, re-admission, and graft restenosis.

### Statistical methods

Continuous variables with normal distribution were expressed as means ± standard deviations (SDs), and those with non-normal distribution were expressed as medians (p25-p75). Categorical variables were expressed as numbers and percentages. Groups were compared using the chi-square test for categorical variables, and the Student’s t-test and Mann-Whitney U test for continuous variables based on their distribution. Multivariate logistic and linear regression analyses were performed to identify independent predictors of postoperative LVEF improvement and absolute LVEF change (ΔLVEF), respectively. Variables with *P* < 0.10 in univariate analysis or with clinical relevance were included in the models, and results were reported as β coefficients with 95% confidence intervals (CIs). All tests were two-tailed, and a value of *P* < 0.05 was considered statistically significant. All analyses were performed using SPSS 20.0 (SPSS, Chicago, IL, USA).

## Results

### Clinical characteristics

The baseline clinical characteristics of the two groups are shown in Table 1. Between November 2018 and July 2019, 156 patients underwent CABG in our hospital. The age of patients in the two groups was comparable. There were no significant differences in crucial clinical characteristics between the two groups.

**Table 1:** Baseline clinical characteristics

Project	M Group (*N* = 78)		C Group (*N* = 78)	*P*
Gender, Female Age [years]		14(17.9%) 63 (56–68)	21(26.9%) 65 (60–70)	0.179 0.078
BMI [kg/m^2^]		25.00 (23.00–27.00)	24.87 (22.76–27.00.76.00)	0.661
Hypertension		55 (70.5%)	55 (70.5%)	0.630
Diabetes		38 (48.7%)	35 (44.9%)	0.957
Hyperlipidemia		29 (37.2%)	30 (38.5%)	0.869
Renal Insufficiency		4 (5.1%)	10 (12.8%)	0.093
Smoking		36 (46.2%)	28 (35.9%)	0.193
Preoperative MI		43 (55.1%)	46 (59.0%)	0.628
Preoperative LVEF [%]		60 (56–64)	60 (46–63)	0.256
Preoperative Troponin T [microg/L]		0.018 (0.010–0.075)	0.014 (0.010–0.044)	0.793
SYNTAX score		28.97 ± 5.04	28.61 ± 4.90	0.648

Values are expressed as mean ± SD, median (interquartile range, 25% to 75%) or n (%). BMI, body mass index; MI, myocardial infarction; LVEF, left ventricular ejection fraction

### Procedural characteristics

The procedural characteristics of the 2 groups are reported in Table 2. There were no significant differences between the two groups regarding the number of grafts (*P* = 0.413) or the average flow (*P* = 0.140). The distribution of coronary target vessels revascularized by venous grafts was comparable between the two groups (*P* > 0.05). However, we found a lower total flow of grafts in the M-group (83.00 [60.75–114.25.75.25] vs. 100.00 [71.00–136.25.00.25] ml/min, *P* = 0.030). The acquisition time (from skin incision to vein removal) of grafts was shorter in the M-group than in the C-group (22.25 [19.63–24.90] vs. 28.00 [25.50–30.60] min, *P* < 0.001), and the PI was lower in the M-group (2.26 ± 0.64 vs. 2.75 ± 0.85 ml/min, *P* < 0.001).

**Table 2:** Procedural characteristics

Project	M Group (*N* = 78)	C Group (*N* = 78)	*P*
Number of Grafts 1 2 3 4 5	2 (2.56%) 19 (24.36%) 30 (38.46%) 26 (33.33%) 1 (1.28%)	4 (5.13%) 12 (15.38%) 29 (37.18%) 31 (39.74%) 2 (2.56%)	0.413
Number of LIMA	25 (32.05%)	28 (35.90%)	0.613
Target vessel			
Right coronary artery	53 (67.95%)	61 (78.21%)	0.149
Diagonal	42 (53.85%)	50 (64.10%)	0.193
Left circumflex	52 (66.67%)	55 (70.51%)	0.605
Total Flow of Grafts [ml/min]	83.00 (60.75–114.25.75.25)	100.00 (71.00–136.25.00.25)	0.030^*^
Average Flow of Grafts [ml/min]	28.00 (20.00–40.00)	32.75 (24.00–41.75.00.75)	0.140
Acquisition time of Grafts [min]	22.25 (19.63–24.90)	28.00 (25.50–30.60)	< 0.001^**^
Pulsatility Index [ml/min]	2.26 ± 0.64	2.75 ± 0.85	< 0.001^**^

Values are expressed as mean ± SD, median (interquartile range, 25% to 75%) or n (%). * *P* < 0.05; ** *P* < 0.01

### Postoperative cardiac function Recovery, Drainage, and complications

Postoperative cardiac function recovery of the two groups is reported in Table 3. There was no difference between the 2 groups regarding troponin T levels on the first three days after surgery (*P* = 0.521, *P* = 0.197, *P* = 0.071). However, we found lower troponin T levels on the 7th day in the M-group (0.19 [0.15–0.21] vs. 0.25 [0.19–0.32] microg/L, *P* < 0.001). The postoperative hospital s5tay time and postoperative LVEF showed no differences between the 2 groups (*P* = 0.181 and 0.491).

**Table 3:** Postoperative cardiac function recovery, drainage and complications

Project	M Group (*N* = 78)	C Group (*N* = 78)	*P*
Troponin T 1 st day [microg/L]	0.92 (0.68–1.17)	0.99 (0.71–1.21)	0.521
Troponin T 2nd day [microg/L]	0.63 (0.56–0.72)	0.66 (0.54–0.79)	0.197
Troponin T 3rd day [microg/L]	0.54 (0.45–0.62)	0.58 (0.50–0.66)	0.071
Troponin T 7th day [microg/L]	0.19 (0.15–0.21)	0.25 (0.19–0.32)	< 0.001^**^
Postoperative LVEF [%]	60 (55–62)	60 (49–60)	0.181
Postoperative Hospital Stay [days]	9 (8–11)	10 (8–13)	0.491
Postoperative drainage in 24 h [ml]	261 (207–316)	243 (207–261)	0.006^**^
Postoperative drainage in 48 h [ml]	374 (311–424)	334 (308–359)	0.001^**^
Postoperative drainage in 72 h [ml]	442 (373–490)	386 (360–412)	< 0.001^**^
Retaking thoracotomy	3(3.8%)	5(6.8%)	0.468
Poor healing of lower limb incision	2(2.6%)	3(3.8%)	0.649
Myocardial infarction (MI)	4(5.1%)	7(9.0%)	0.348
Atrial fibrillation (AF)	18(23.1%)	20(25.6%)	0.709
Perioperative death	1(1.3%)	2(2.6%)	0.560

Values are expressed as mean ± SD, median (interquartile range, 25% to 75%) or n (%). LVEF, left ventricular ejection fraction. * *P* < 0.05; ** *P* < 0.01

The postoperative drainage and complications of the two groups are reported in Table 3. Patients in the M-group had more postoperative drainage in the first 72 h (*P* = 0.006, *P* = 0.001, *P* < 0.001). Retaking thoracotomy occurred in 3 patients (3.8%) in the M-group and 5 patients (6.8%) in the C-group (*P* = 0.468). Poor healing of lower limb incisions occurred in 2 patients (2.6%) in the M-group and 3 patients (3.8%) in the C-group (*P* = 0.649). Perioperative death occurred in 1 patient (1.3%) in the M-group and 2 patients (2.6%) in the C-group (*P* = 0.560). Myocardial infarction (MI) occurred in 4 patients (5.1%) in the M-group and 7 patients in the C-group (*P* = 0.348). Atrial fibrillation (AF) occurred in 18 patients (23.1%) in the M-group and 20 patients (25.6%) in the C-group (*P* = 0.709).

### Follow-up

The follow-up data are presented in Table 4. Follow-ups were performed for at least 24 months. Except for three death cases (one in the M-group and two in the C-group), all patients underwent relevant examinations. Echocardiography was used to assess cardiac function, and coronary artery CTA was used to assess the condition of the patient’s grafts. At 24 months, LVEF was higher in the M-group (65 [62–67] vs. 60 [55–63] %, *P* < 0.001). Multivariable linear regression identified the MNT technique as an independent factor associated with postoperative improvement in LVEF at 24 months (β = 8.661, 95% CI 6.890–10.432, *p* < 0.01) (Fig. S1). Lower extremity edema (9.1% vs. 17.1%, *P* = 0.141), lower extremity numbness (5.2% vs. 11.8%, *P* = 0.140), and angina pectoris (9.1% vs. 14.5%, *P* = 0.301) occurred in both groups, but there was no significant difference between them. Both re-admission (5.2% vs. 11.8%, *P* = 0.236) and graft restenosis (6.5% vs. 14.5%, *P* = 0.107) occurred more frequently in the C-group, although there were no significant differences between the groups.

**Table 4:** Follow up

Project	M Group (*N* = 77)	C Group (*N* = 76)	*P*
LVEF 24 months [%]	65 (62–67)	60 (55–63)	< 0.001^**^
Lower extremity edema 24 months	7 (9.1%)	13 (17.1%)	0.141
Lower extremity numbness 24 months	4 (5.2%)	9 (11.8%)	0.140
Angina pectoris 24 months	7 (9.1%)	11 (14.5%)	0.301
Re-admission 24 months	4 (5.2%)	9(11.8%)	0.236
Grafts restenosis 24 months	5 (6.5%)	11 (14.5%)	0.107
Cardiac death 24 months	0 (0%)	0 (0%)	/
Non-cardiac death 24 months	0 (0%)	0 (0%)	/

Values are expressed as mean ± SD, median (interquartile range, 25% to 75%) or n (%). LVEF, left ventricular ejection fraction. * *P* < 0.05; ** *P* < 0.01

### Temperature measurement

**Fig. 2:**
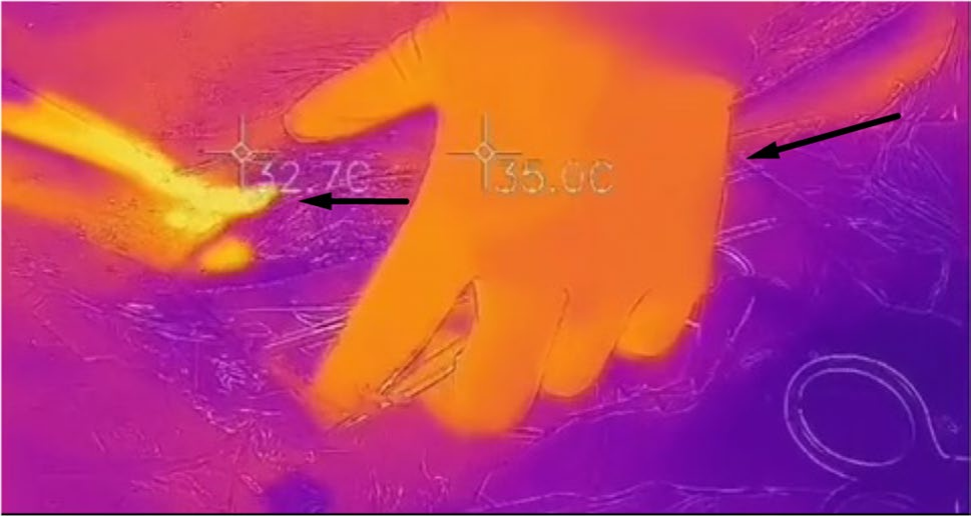
Measurement of the ambient temperature of harmonic scalpel. This photograph shows the temperature around the grafts. Although the harmonic scalpel may generate high temperature, it didn’t affect the ambient temperature around the SV. The black arrows show the orientation of the SV

The temperature around the grafts is shown in Fig. 2. The high temperature generated by the harmonic scalpel did not affect the temperature around the SV (32.7 °C). The black arrows show the orientation of the SV.

### Histomorphology and immunohistochemistry

**Fig. 3:**
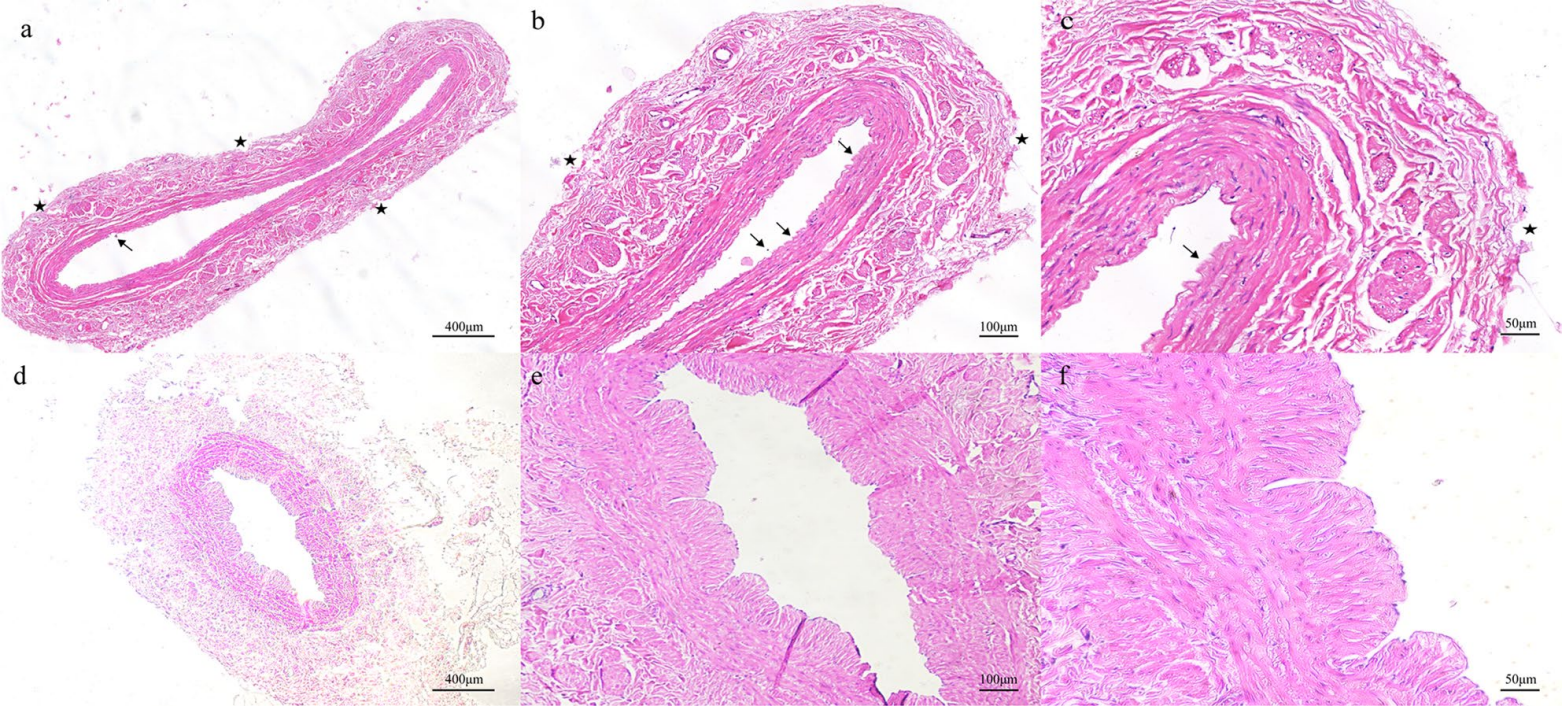
Hematoxylin and eosin (H&E) staining of cross-sections of the human SV harvested by MNT and conventional technique. Histological presentation of the vein samples obtained by two different techniques shows focal defects of the endothelium (arrows) and adventitial layer with focally absent vasa vasorum (stars) of C-group vein samples (**A**, **B**, **C**), and intact vein wall of M-group vein samples (**D**, **E**, **F**)

The comprehensive conditions are shown by the cross-sections of human SV stained with H&E stain in Fig. 3. As seen in the graphs, the venous vessels harvested by conventional technique demonstrate structural damage from the conventional surgical procedure, including endothelial cell rupture, basement membrane tears, vasa vasorum angiorrhexis, vessel wall structural failure, as well as surrounding connective tissue and adipose tissue loss. The photomicrographs of H&E-stained cross-sections harvested by MNT technique contrast with the C-group cross-sections, showing almost perfect vessel wall structures including endothelium, basement membrane, and supporting tissue integrity.

**Fig. 4:**
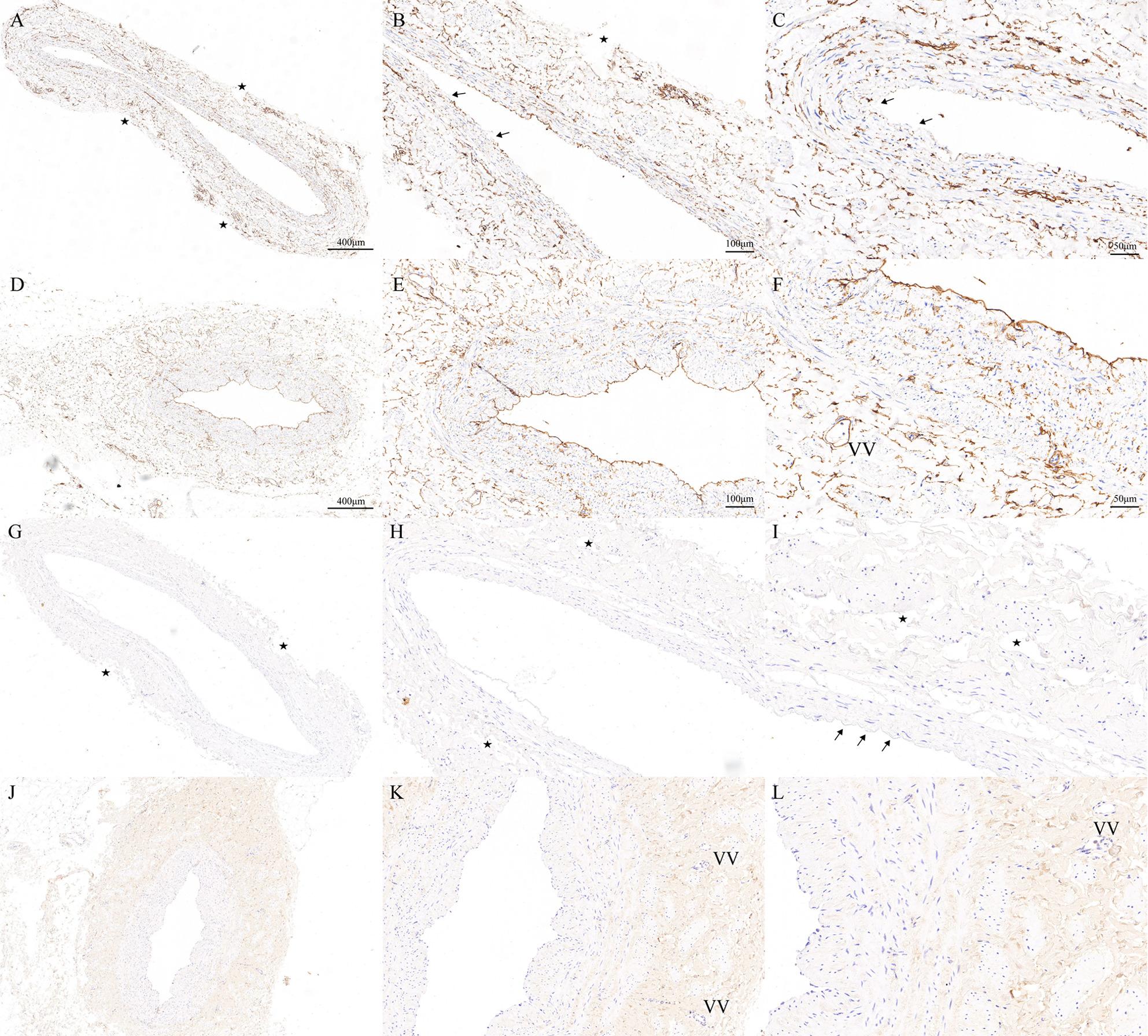
Cross-sections of the human SV harvested by MNT and conventional technique stained with Immunohistochemical (IHC) stain highlighting CD34 and Collagen I in tissue. CD34 IHC staining shows focally minimal staining of the endothelium (arrow) and defects of the adventitial layer (star) of C-group vein samples (**A**, **B**, **C**), in contrast with strong staining (brown colored) of the endothelium and vasa vasorum (VV) in the adventitial layer of M-group vein samples (**D**, **E**, **F**). Collagen I IHC staining shows focal defects of the endothelium (arrows) and the wall integrity (stars) of C-group vein samples (**G**, **H**, **I**), in contrast with intact endothelium and vasa vasorum (VV) in the adventitial layer of M-group vein samples (**J**, **K**, **L**)

To further demonstrate the results, the cross-sections from both conventional and MNT techniques were stained with special stains such as IHC stains. All the special stain results were highly consistent with the H&E-stained cross-sections as shown in Fig. 3. The IHC (CD34) stained sections demonstrate the basement membranes of the endothelium which are colored dark-brown to black. As shown in Fig. 4(A-F), most of the basement membranes are missing in the SV sections harvested by conventional technique, while in sharp contrast to the C-group, basement membranes display close to perfect continuity and integrity in the SV sections harvested by MNT technique. As shown in Fig. 4(G-L), collagen I was targeted by IHC stain to highlight the vessel wall structural integrity. The results suggest a significant difference between conventional and MNT techniques, with a great improvement in vessel wall preservation in the M-group.

## Discussion

While arterial grafts like the IMA and RA demonstrate superior long-term patency in CABG, the SV remains widely used due to its accessibility [14, 15]. The low long-term patency rate of the SV graft is not only because of the characteristics of the vein itself but also due to damage caused by improper harvesting techniques [16].

The method of harvesting the saphenous vein has received increased attention in recent years. Since 1993, Souza [13] used the NT technique to harvest the saphenous vein for the first time and conducted a longitudinal study. Souza [17] reported that one hundred fifty-six patients who underwent CABG were randomized to three saphenous vein harvesting groups, and the patency of the SVG in the NT group was higher than that of the traditional method (95% vs. 89%) at 18 months mean follow-up time. An eight-year follow-up study found that the patency rate of SVG harvested using the NT technique was comparable to that of the RA [18]. Recent evidence has further suggested that the NT vein is not merely comparable to the radial artery—it may even surpass it. Ferrari et al. conducted a large-scale analysis involving more than 1,500 consecutive patients and reported superior long-term patency of NT vein grafts compared with conventional grafts, showing outcomes comparable to those of IMA [19]. These findings reinforce the concept that, when appropriately harvested, the NT vein can serve as a highly durable conduit, potentially expanding its role in elderly or high-risk patients for whom the use of arterial grafts, such as the IMA, may be limited. The NT technology was recommended as a Class IIa level in the 2018 European Society of Cardiology (ESC) and the European Association for Cardio-Thoracic Surgery (EACTS) myocardial vascular reconstruction guidelines [20]. In addition to Sweden, more cardiac surgeons in Brazil, South Korea, Japan, and China have begun to use this technique [21–23]. Despite endorsement by ESC/EACTS guidelines, NT technique’s adoption remains limited globally, partly due to technical complexities like prolonged intraoperative graft inspection and increased postoperative drainage or hematoma risks [24].

Extravascular stenting has been explored to enhance venous graft patency by mitigating wall stress from arterial hemodynamics, thereby reducing endothelial injury and smooth muscle hyperplasia [25, 26]. Early studies usually used ordinary metal stents to limit the excessive expansion of grafts, but studies have found that chronic vascular stimulation resulted in accelerated intimal hyperplasia, vascular smooth muscle proliferation and migration, and re-stenosis of grafts [27, 28]. Based on this situation, recent innovations include porous, biodegradable, drug-eluting, and conductive electroporation stents, yet material-induced inflammation and uncertain long-term efficacy remain barriers to clinical translation [29–31].

In contrast, the fat on the surface of the pedicled great saphenous vein and the surrounding connective tissue has a natural protective effect on the grafts [23, 32, 33]. In addition to nourishing grafts and maintaining vascular dilation, it can also limit the excessive expansion of the grafts, reduce vascular endothelial injury caused by arterial pressure, reduce proliferation and inward migration of medial smooth muscle, prevent graft stenosis, and improve long-term patency rate [24, 34, 35]. This method provides another option for improving the expected patency rate of venous grafts. Based on the above considerations, we ultimately determined the method of harvesting the pedicled SV with a harmonic scalpel after reviewing the data and preliminary experiments.

In our study, the acquisition time of grafts was shorter in the M-group, due to the avoidance of pressurized water pumping to check grafts. As a medical instrument routinely used in clinical practice, the working principle of the harmonic scalpel is to make the metal knife head oscillate mechanically at the ultrasonic frequency of 55.5 kHz through the ultrasonic frequency generator, vaporizing water molecules in tissues, breaking the hydrogen bonds of proteins, disintegrating cells, cutting or coagulating tissues, and closing blood vessels. Regarding potential high-temperature damage to the grafts’ wall cells during harmonic scalpel cutting, we first measured the temperature around the grafts with an infrared thermometer during the harvesting process. Due to the harmonic scalpel’s cutting speed and cutting position (approximately 5 mm away from the grafts’ wall), there was no significant temperature rise around the grafts. The graft flow in the M-group was lower, which may be primarily attributed to the fact that the veins in the M-group did not undergo excessive dilation, resulting in a smaller early-stage graft diameter compared to the C-group. However, the M-group exhibited better PI than the C-group. PI is considered an important indicator of the quality of the target vessel, the quality of the grafts, and the quality of the anastomosis [36]. This indicates that the SVG harvested by MNT has good patency when there is no difference in target vessel conditions.

The NT technique preserves graft structural integrity and endothelial NO production by retaining perivenous adipose and microvasculature. Studies confirm elevated endothelial nitric oxide synthase (eNOS) expression in NT-harvested grafts, correlating with reduced intimal hyperplasia and restenosis [23]^,^ [37, 38]. Another study showed that the expression of eNOS in the NT group was 35.5% higher than that in the conventional harvesting group [38]. Dashwood MR [39] compared two methods of harvesting grafts and estimated the damage of the SVG by observing the thickness of the vessel wall, finding that the NT group had higher endothelial integrity and higher eNOS expression. Beyond eNOS, perivascular tissue in NT grafts may mitigate inflammation and mechanical distortion [40]. In our study, most of the basement membranes are missing in the SV sections harvested by conventional technique, while in sharp contrast to the C-group, basement membranes display close to perfect continuity and integrity in the SV sections harvested by MNT technique. Additionally, collagen I staining suggests a great improvement in vessel wall preservation in the M-group. There was no obvious abnormality in the morphology of vascular endothelial cells and smooth muscle cells, which proved that the grafts were not damaged by the use of the harmonic scalpel.

Regarding postoperative cardiac function recovery, we found that the troponin T level on the 7th day was lower in the M-group, suggesting that MNT technique may effectively accelerate the recovery of heart function. The difference between the 2 groups could also be partly due to reduced graft dilatation. The postoperative drainage in the first 72 h after surgery was higher in the M-group. It is possible that the MNT technique failed to completely close some small branches, but this didn’t affect the overall prognosis. No bleeding due to unclosed side branches was found in patients with postoperative bleeding requiring reoperation. Therefore, the reason for the higher postoperative drainage in the M-group will be further investigated in a subsequent study. We found that the frequency of wound complications in the M-group was not only lower than that in the C-group but also lower than that reported in previous studies [41, 42]. The reason may be that using the harmonic scalpel can close many small venous branches during the procedure, resulting in less bleeding from the wound after surgery, thus not affecting wound healing. Additionally, we tried to avoid damaging surrounding tissues when harvesting the SV. Before surgery, the SV and its important branches were marked on the skin with the help of doppler ultrasonography, minimizing damage to surrounding tissue, which can decrease the effect on postoperative wound healing.

Another benefit of MNT technique is significantly improved recovery of cardiac function during follow-up. To eliminate potential confounding factors, we performed a multivariable linear regression analysis including age, sex, diabetes, hypertension, dyslipidemia, smoking history, SYNTAX score, and baseline LVEF as covariates. The MNT technique remained an independent predictor of postoperative LVEF improvement (β = 8.661, 95% CI 6.890–10.432, *p* < 0.01).These findings suggest that the beneficial effect of the MNT technique is not attributable merely to differences in the distal coronary bed condition or patient selection, but reflects the biological advantages of preserving the vein’s endothelial integrity and perivascular microstructure during harvesting. At 24 months postoperatively, the LVEF in the M-group was higher. Although no significant difference in graft patency was detected at the two-year follow-up, this may be attributed to the sample size and follow-up duration. We anticipate that with a longer follow-up period, the difference will become more apparent.

The study was limited by the small sample size; an extensive, multi-center study is necessary to confirm the findings. Additionally, the follow-up period was not sufficiently long. Also, coronary artery CTA measurements are less accurate compared with conventional angiography. In the future, we may need to include more detailed evaluation criteria, such as intravenous ultrasound (IVUS) and myocardial nuclear imaging. Furthermore, certain anatomical and procedural parameters—including the diameters of the saphenous veins, degree of stenosis in coronary lesions, and the presence of sequential lesions—were not systematically recorded in this study, which may limit the comprehensive assessment of factors influencing graft performance and cardiac function recovery. Finally, future studies directly comparing two no-touch techniques—with and without the use of a harmonic scalpel—are warranted to further determine the independent role of the device.

## Conclusions

In summary, the technical features of the SV graft harvesting technique involved in this study are based on the core concepts of vascular protection and reduction of complications. The results of the study were twofold. First, it was again demonstrated that using the MNT technique to harvest SVG for CABG results in good short-term and middle-term patency. More importantly, it was confirmed that the modified harmonic scalpel technique allowed the NT technique to make the acquisition process easier and faster, and may contribute to improve the recovery of cardiac function. It is hoped that this modified harmonic scalpel technique may provide a useful reference for optimizing the no-touch harvesting technique in future studies.

## Supplementary Information

Supplementary Material 1

## Data Availability

The raw data supporting the conclusions of this article will be made available by Ge Zhu (Email: zhuge2011083@gmail.com), waithout undue reservation.

## Abbreviations

SVG: Saphenous vein grafts
CHD: Coronary heart disease
CABG: Coronary artery bypass grafting
LVEF: Left ventricular ejection fraction
IMA: Internal mammary artery
RA: Radial artery
GA: Gastroepiploic artery
SV: Saphenous vein
MNT: Modified “no-touch” harvesting technique
