# Supplementary material for: Short- and mid-term clinical outcomes of harmonic scalpel-assisted no-touch technique of the saphenous vein grafts harvesting in coronary bypass grafting
Source: J Cardiothorac Surg. 2025 Dec 30;21:58. doi: 10.1186/s13019-025-03823-x (PMC12860025; doi:10.1186/s13019-025-03823-x)
Supplement: Supplementary file 1 — Supplementary Material 1 [file 13019_2025_3823_MOESM1_ESM.docx]

**Short- and Mid-term Clinical Outcomes of a Modified No-touch Technique of the Great Saphenous Vein Grafts Harvesting in CABGs**

**Running title:** Outcomes of a Modified No-touch Technique

Ge Zhu^†^, Su Wang^†^, Chenjun Han, Qiang Liu, Jian Zhou^*^, Wangfu Zang^*^

Department of Cardio-Thoracic Surgery, Shanghai Tenth People’s Hospital, School of Medicine, Tongji University, Shanghai, China

† These authors contributed equally to this work and share first authorship.

**E-mails:**

Ge Zhu: Zhuge2011083@gmail.com

Su Wang: wangsu0404@qq.com

Chenjun Han: hanson1163@163.com

Qiang Liu: liuqiang5233@163.com

Jian Zhou: drjose@163.com

Wangfu Zang: zangwf@tongji.edu.cn

***Corresponding author:**

Wangfu Zang, Department of Cardio-Thoracic Surgery, Shanghai Tenth People’s Hospital, School of Medicine, Tongji University, Shanghai, China

Email: zangwf@tongji.edu.cn, Tel: +86 18917687413

Jian Zhou, Department of Cardio-Thoracic Surgery, Shanghai Tenth People’s Hospital, School of Medicine, Tongji University, Shanghai, China

Email: drjose@163.com, Tel: +86 18917687427


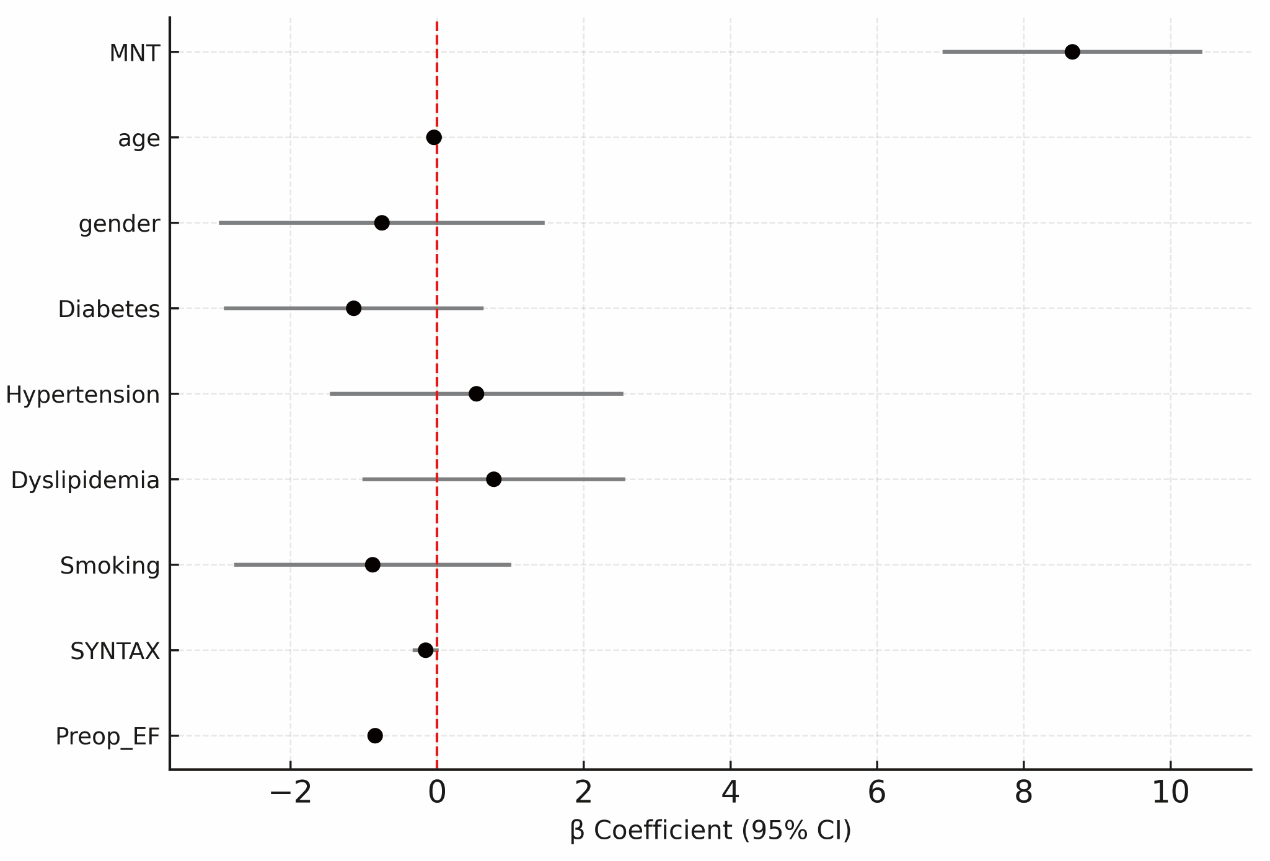


**Fig. S1** Multivariable linear regression identifying independent predictors of LVEF change at 24 months after CABG. The red dashed line indicates the null value (β = 0).

**Table S1** Multivariable linear regression analysis of predictors associated with postoperative LVEF change at 24 months.

| **Project** | **β (Coefficient)** | **95% CI** | **P** |
| --- | --- | --- | --- |
| MNT | 8.661 | 6.890 – 10.432 | <0.01^*^ |
| Age | -0.042 | -0.152 – 0.068 | 0.451 |
| Gender | -0.754 | -2.973 – 1.465 | 0.503 |
| Diabetes | -1.136 | -2.907 – 0.636 | 0.207 |
| Hypertension | 0.538 | -1.461 – 2.538 | 0.595 |
| Dyslipidemia | -0.491 | -2.582 – 1.600 | 0.648 |
| Smoking | -0.716 | -2.979 – 1.548 | 0.536 |
| SYNTAX score | -0.084 | -0.241 – 0.072 | 0.283 |
| Preoperative EF | -0.192 | -0.258 – -0.125 | <0.01^*^ |
